# Investigation of a human brucellosis outbreak in Douz, Tunisia, 2018

**DOI:** 10.4178/epih.e2022048

**Published:** 2022-05-18

**Authors:** Nejib Charaa, Rabaa Ghrab, Aicha Ben Othman, Mohamed Makhlouf, Hejer Ltaief, Nissaf Ben Alaya, Mohamed Chahed

**Affiliations:** 1 Preventive Health Division, Regional Directorate of Health, Kebili, Tunisia; 2 Douz Health District, Douz, Tunisia; 3 Preventive Health Division, Regional Directorate of Health, Sfax, Tunisia; 4 National Observatory of New and Emerging Diseases, Ministry of Health, Tunis, Tunisia; 5 Departement of Preventive Medicine and Epidemiology, Faculty of Medicine of Tunis, Tunis, Tunisia

**Keywords:** Brucellosis, Outbreaks, Case-control studies, One Health, Zoonoses, Foodborne disease

## Abstract

**OBJECTIVES:**

In 2017, the incidence of human brucellosis in Tunisia was 9.8 per 100,000 population. In the Douz district, 2 cases were reported in March 2018. Prior to that date, the last indigenous cases to be reported in Douz had been in 2015. This study aimed to identify the source of this new contamination and recommend control interventions.

**METHODS:**

This case-control study included residents of Douz who presented with clinical symptoms of brucellosis and had a subsequent Wright test antibody titer ≥ 1/160. The controls were neighbors of the infected cases who had a negative Rose Bengal test. Univariate and multivariate analyses were performed to estimate the odds ratios of risk factors. Goats belonging to the cases and controls were actively screened.

**RESULTS:**

Twenty-five infected cases and 52 uninfected controls were enrolled. All infected cases had consumed goat milk and 92% had purchased it from the same breeder. Consumption of goat milk from this breeder (adjusted odds ratio [aOR], 30.78; 95% confidence interval [CI], 6.47 to 235.91) and overall consumption of raw goat milk (aOR, 14.84; 95% CI, 2.04 to 310.44) were independent risk factors for brucellosis. The breeder had 18 goats, 5 of which were smuggled from a neighboring country. Three of those goats were diagnosed with brucellosis.

**CONCLUSIONS:**

Consumption of raw milk from smuggled sick goats was the main risk factor in this outbreak. The sick goats were slaughtered and an education campaign was conducted. Vaccination, control of cross-border animal movements, and control of goat milk sales must be strengthened to prevent the spread of brucellosis in southwestern Tunisia.

## GRAPHICAL ABSTRACT

[Fig f3-epih-44-e2022048]

## INTRODUCTION

**Figure 1.**
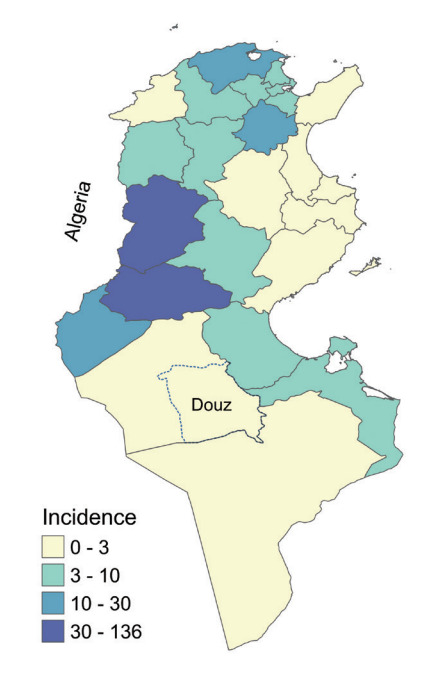
Incidence of human brucellosis per 100,000 population, Tunisia, 2017 (n=1,124).

Brucellosis is a bacterial zoonosis caused by infection with bacteria of the genus *Brucella*. The *Brucella* species most frequently responsible for human infections are *B. melitensis*, *B. abortus*, and *B. suis*. The disease reservoir consists of goats and sheep for *B. melitensis*, cattle for *B. abortus*, and pigs for *B. suis* [1]. *B. melitensis* is the most virulent and most frequently isolated species in humans [2]. Laboratory diagnosis of brucellosis includes isolation of *Brucella* from a blood or bone marrow culture and serologic detection of specific serum antibodies [3]. Many diagnostic tests have been developed such as the enzyme-linked immunoassay agglutination test, the Rose Bengal test, and the serum agglutination test or Wright test, which is the test most commonly used to diagnose human brucellosis [4]. For rapid screening, the Rose Bengal test can be used due to its excellent sensitivity [3]. Transmission of brucellosis is through direct contact with livestock or indirectly through the consumption of raw milk or unpasteurized milk by-products [2]. Human epidemiology is closely associated with animal epidemiology [3]. Brucellosis has high epidemic potential when a population is exposed to a common source of infection [4] and is the most common zoonotic infection in the world, with more than 500,000 new cases each year [2]. Its incidence in endemic areas varies widely, from < 0.01 to > 200 per 100,000 population [5]. It has become rare in countries that have instituted vaccination policies to eradicate the disease in animals [6,7]. Despite under-reporting and the scarcity of epidemiological data, it is recognized that the disease is endemic in Africa [8]. In Tunisia, the overall incidence was 9.8 per 100,000 population in 2017 [9]. In the Douz district prior to 2018, the last reported indigenous cases had been in 2015 (Figure 1).

On Monday, March 19, 2018, an 11-year-old girl and a 57-year-old woman were hospitalized at hospital in Douz for human brucellosis. The preliminary investigation revealed that they were from the same neighborhood, that they consumed goat milk bought from the same breeder, and that the father of the young girl had been hospitalized in Sfax for brucellosis. The epidemic threshold set by the national intervention epidemiology guide [10] is at least 2 grouped cases. An outbreak was therefore confirmed and declared. The regional health surveillance unit conducted epidemiological and veterinary investigations around these 2 cases.

The objectives of these investigations were to describe the epidemic focus, identify the source of contamination, set up immediate control interventions, and propose further prevention measures.

## MATERIALS AND METHODS

### Study area

The city of Douz is a municipality located in southwest Tunisia that had 30,245 inhabitants in 2016 (Figure 1). The economy of the city is based on tourism, the production of dates, and the breeding of goats, sheep, and camels. Goat breeding in the home is usual in the city. Goat milk is essential food source for many families.

### Epidemiological investigation

#### Study design

A case-control epidemiological study was conducted by local health professionals, trained and supervised by a field epidemiologist. The study period was January through March 2018. The researchers agreed to investigate at least 2 controls for each case. The required sample size estimated using the Epicalc R package was 60 people (40 controls and 20 cases), assuming 15% exposure in controls, 80% power to detect an odds ratio (OR) of 5, and an alpha error of 5%. The cases were recruited from an active case index. Controls were selected from patients consulting the local health center in the same neighborhood as the cases between March 23 and 28, 2018. All patients who met the definition of a control and agreed to participate in our investigation were selected.

#### Case/control definition

Case: A case was a resident of Douz who presented from January through March 2018, with at least 2 of the following signs: fever, night sweats, arthralgia, asthenia, headache, and anorexia, and who also had a positive Wright antibody agglutination serology titer ≥ 1/160.

Control: A control was as a neighbor of the case who had a negative Rose Bengal test and who showed no clinical signs of brucellosis.

#### Data collection

Data were collected using a standardized form. Socio-demographic, clinical, and risk exposure information was collected. Two main types of exposure were explored: consumption of dairy products and contact with animals. To determine exposure, the questionnaire covered the 3 months preceding the onset of symptoms.

### Veterinary investigation

As part of the national One Health plan, all identified cases were reported to a veterinarian, who conducted a further investigation to identify animal cases of brucellosis among the flocks. Blood samples were taken from all goats for serological screening using the Rose Bengal Test and preventive measures were implemented to control the outbreak.

### Statistical analysis

Databases were analyzed on the R environment version 1.2.1335 (RStudio, Boston, MA, USA). The relationships between variables were measured as the OR of risk factors, calculated with their confidence intervals (CI) using a univariable analysis at the α threshold of 5% using the epiR package (0.9-93). Statistical significance for all tests was indicated by a p-value < 0.05. To eliminate the effect of confounding factors, only variables for which the OR in the univariable analysis had a p-value < 0.05 were included in the multivariate logistic regression model.

### Ethics statement

The investigation of this outbreak was made within the framework of the mission granted to the regional health monitoring unit, therefore no approval was necessary.

## RESULTS

### Descriptive study

#### Socio-demographic characteristics

The study population consisted of 77 participants. During the study period, 30 human brucellosis cases were reported to the mandatory notification system, but only 25 cases met the case definition and were enrolled in the study. Fifty-two controls were also recruited. The median age of the participants was 43 years: 41 years (range, 3 to 89) for cases and 43 years (range, 6 to 91) for controls. The male/female ratio of the total study population was 0.5 (Table 1).

#### Risk factors

All 25 cases in the study consumed goat milk. They reported that this milk came from 3 sources. Two sources were breeders who owned goats for personal consumption. The third was a breeder who sold milk door-to-door to 17 families unaffiliated with a commercial store. Twenty-three of the cases in our study (92%) drank milk purchased from this breeder. All cases belonged to 9 families. The family of this breeder was the strongly most affected, with 6 cases. All cases consumed goat milk or goat-milk products (fresh curd or fermented milk) daily or frequently. Only 1 case said that he had consumed boiled milk. Of the total 25 cases, 11 had frequent contact with goats in general and 5 cases had contact with the goats of the breeder who sold his milk door-to-door.

#### Clinical features

Of the 25 cases, 8 were hospitalized. Children under 8 years of age were treated with trimethoprim/sulfamethoxazole and rifampicin. Adults and children over 8 years of age were treated with doxycycline and rifampicin. The diagnosis delay ranged from 1 day to 30 days (median, 10). The first 3 cases received late diagnoses. The clinical picture of the first case was atypical; in addition to the clinical signs of brucellosis, the patient presented with mental confusion suggestive of encephalopathy or meningitis. Fever, asthenia, headache, and night sweats were present in 84%, 76%, 72%, and 64% of patients, respectively.

#### Epidemic curve and spatial distribution of cases

**Figure 2.**
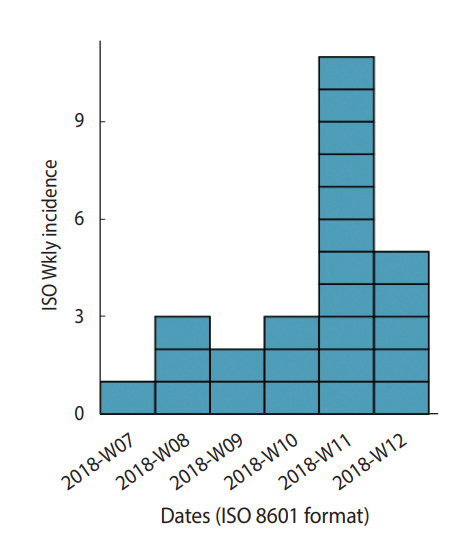
Weekly epidemic curve of human brucellosis cases, Douz, Tunisia, March 2018 (n=25). ISO, International Organization for Standardization.

An epidemic curve was established based on the date that patients reported their clinical signs first appeared. The weekly epidemic curve highlighted an epidemic peak in the 11th week of 2018. The first case presented with clinical signs on February 18, 2018, and the last case on March 30, 2018. The approximate median incubation time was 41 days, which corresponds to the extent of the epidemic. The approximated maximum exposure was at week 5, between late January and early February. The shape of the curve suggested a common point source. The distribution of cases showed that they all resided in the same neighborhood, within a 500 m perimeter around the goat breeder who sold his milk (Figure 2).

### Analytic study

The most significant risk factor highlighted by the univariate analysis was the consumption of goat milk purchased from the breeder who sold his milk door-to-door (OR, 54.94; 95% CI, 10.94 to 275.88). The odds of consuming it raw were 108 times greater in cases than in controls (OR, 108.10; 95% CI, 19.47 to 600.07). The odds of consuming raw goat milk from any origin were 35 times greater in cases than in controls (OR, 35.43; 95% CI, 4.45 to 282.36). Although contact with the goats of this breeder was associated with the occurrence of brucellosis in the univariate analysis (OR, 12.75; 95% CI, 1.40 to 116.04), the multivariate logistic regression showed that only consumption of goat milk from the breeder who sold his milk door-to-door (adjusted odds ratio [aOR], 30.78; 95% CI, 6.47 to 235.91) and consumption of raw goat milk in general (aOR, 14.84; 95% CI, 2.04 to 310.44) were independently related to the occurrence of brucellosis in these cases. The risk factor of contact with the goats of this breeder was statistically insignificant (aOR, 1.39; 95% CI, 0.17 to 29.85) (Table 2).

### Veterinary investigation

The 3 goat herders were visited by the veterinarian and the environmental health team. The first 2 breeders had 3 goats and 7 goats, respectively. The breeder selling his milk door-to-door had 18 goats, 5 of which had been illegally imported from Algeria within the past 3 months. All goats were subsequently vaccinated against brucellosis except for the 5 smuggled goats. Serological screening revealed that 3 of the 5 smuggled goats had the disease.

### Control measures

Following the preliminary investigation, which began on the day of the call about the 2 index cases (March 19, 2018), the only epidemiological link found was the consumption of goat milk from a single breeder. Therefore, the potential source of the outbreak was identified. The breeder (owner of the source farm of the outbreak) was contacted the same day to inform him of the probable existence of sick goats among his herd, to prohibit him from selling and consuming the milk of his goats, and to make a list of his clients. Between March 19 and 24, 2018, active screening for human brucellosis among all members of the breeder household and all his clients was carried out. At the same time, a case-finding survey was conducted among all doctors in the Douz district. The veterinarian was officially informed on March 20, 2018, and obtained blood samples that same day from all the goats for serological screening. The sick goats were sequestered and isolated, the sites were disinfected, and the products and materials likely to be contaminated were destroyed. Moreover, other animals on the farm were placed under surveillance. An education campaign for the public was carried out in April 2018, to emphasize the importance of consuming only sterilized milk.

## DISCUSSION

In March 2018, several cases of brucellosis were reported in the Tunisian city of Douz. Epidemiological and veterinary investigations were conducted to identify the source of contamination and propose appropriate control and prevention measures. The investigation team conducted a case-control study. Twenty-five of the 30 human brucellosis cases reported to the mandatory notification system were included in the study. Five cases were excluded because they did not meet the specific case definition. The presence of clinical signs and a Wright serologic agglutination antibody titer ≥ 1/160 was required to admit cases to the study. Although the case definition was very specific in our study, positive serology with the presence of clinical signs and/or exposure to the source may have been sufficient to identify cases. The recommendations of the Tunisian Ministry of Health [11], as well as other authors [12], consider an epidemiological context with evidence of exposure to *Brucella* to be of high diagnostic value. Fifty-two controls were selected from neighbors of the study cases, including those who consulted the local health center and showed no clinical signs of brucellosis. A negative Rose Bengal test was required to classify an individual as a control. This test is considered highly reliable in the diagnosis of human brucellosis [13]. The sample size was adequate for detecting significant differences between cases and controls.

All infected cases consumed goat milk and 23 had purchased milk from the same breeder. The most important risk factor identified by the univariate and multivariate analyses was the consumption of raw milk from the goats of this breeder. His herd contained 18 goats, of which 5 had been smuggled from Algeria within the past 3 months. Three of the smuggled goats were diagnosed with brucellosis. They were isolated for slaughter. The study cases belonged to 9 households, and 6 cases were family members of the breeder who owned the source herd. Some studies consider livestock ownership [14] and the existence of a family member infected with brucellosis [15] to be risk factors for the disease. A health education campaign was conducted to prevent the use of domestic raw milk. The results of our study were inconsistent with those of Khamassi Khbou et al. [16], in the Gafsa district, which found that consumption of raw milk and its derivatives was not a risk factor for brucellosis infection, while the handling of ruminant females during abortion or parturition was the main risk factor. Data from several sources identify work with animals as the primary risk factor [17]. A human brucellosis outbreak among agricultural workers in Argentina was investigated and revealed a close association with an epidemic of goat abortions that had recently occurred on the same farm [18]. Other studies have shown that the consumption of raw and unpasteurized dairy products is the main risk factor for brucellosis infection [19]. Results of an Italian study indicate that brucellosis in Italy is primarily a foodborne zoonosis rather than an occupational disease [5].

The sex ratio (male/female) among the 25 cases was 0.8 and the median age was 41 years. This female predominance has also been reported by other studies [14,20]. However, Khamassi Khbou et al. [16], in their 2015 study of human brucellosis cases in the Gafsa district, and Aloufi et al. [21] in Saudi Arabia reported that young males in contact with ruminants were at highest risk of contracting brucellosis. Male predominance was explained by the fact that raw milk was consumed more frequently by men in a Libyan study [22]. Corbel et al. [3] explained the differences between the 2 sexes by the fact that, in countries where food hygiene prevents foodborne brucellosis, the disease is predominantly occupational, and most cases are in male aged 20-45 years. In countries where it is difficult to apply hygiene measures to the management of goat milk products, the entire population can be affected by the disease.

The weekly epidemic curve showed a peak between Monday, March 12, 2018, and Sunday, March 18, 2018. The maximum exposure was estimated to be at week 5 (late January to early February). Many studies report a significant seasonal variation in the incidence of acute brucellosis, with most cases occurring in spring and summer [20,21], coinciding with the peak period for abortions and parturitions among farm animals. This peak results in the highest level of exposure for those who work with animals and those who consume their milk [3].

Although the majority of studies have found that brucellosis is a rural disease [23,24], the distribution of cases in our study by place of residence showed that all resided in an urban district. The source farm of the outbreak was in this same neighborhood. Goat farming in a family residence is not unusual in Douz. The consumption of goat milk is an essential source of nutrition for many families. Sheep and goats are the most important domestic animals in many developing countries [12].

Of the 25 cases, 8 were hospitalized. The patients were treated according to recommendations from the Tunisian Ministry of Health (Directorate of Basic Health Care) [11] which suggest that optimal treatment for uncomplicated brucellosis should be based on a 6-week course of doxycycline, combined with streptomycin for 2-3 weeks, or rifampicin for 6 weeks. We did not find in these recommendations prophylactic treatment for cases of exposure to the disease risk factors. The median time to diagnosis was 10 days with a maximum of 30 days for the first patient whose clinical picture was atypical. The clinical diagnosis of brucellosis is difficult since it mimics other febrile diseases and is often misdiagnosed [25].

In Tunisia, 1,124 cases were recorded in 2017 [26]. The Kasserine and Gafsa regions were the most affected with 223 and 471 cases, respectively. In the governorate of Tozeur, the number of brucellosis cases increased from 7 cases in 2015 to 32 cases in 2017. Kebili, located in the southwest area bordering Gafsa and Tozeur, has had a lower incidence in recent years [26]. According to data from the Kebili regional health directorate, 8 cases were reported in 2016 and only 2 cases in 2017 throughout the governorate. The outbreak in our investigation identifies a possible extension of the disease into the southwest region. These governorates occupy central and southwestern Tunisia on the Algeria-Tunisia border. Several economic and security reports confirm animal smuggling between Algeria, Libya, and the governorates of west central and southern Tunisia [27]. The uncontrolled transport of animals across borders and between different regions of the same country increases the risk of propagating brucellosis [28]. North African countries are vulnerable to several transboundary animal diseases, including brucellosis, because of their geographical location and their borders with the Sahel region [29]. Algerian and Libyan studies confirm the endemic state of the disease in goat herds in the eastern region of Algeria and the northwestern region of Libya [22,30]. The *Brucella* strains of the Maghreb (Morocco, Algeria, and Tunisia) were grouped into 2 geographical lineages, suggesting the existence of an indigenous group from the Maghreb and a line resulting from socio-historical links with Europe [31].

The measures taken by the intervention team to control this epidemic were based on directives from the Directorate of Basic Health Care [11] and the Decree of July 14, 2009, establishing the nomenclature of regulated animal diseases and enacting the general measures applicable to these diseases [32]. Additional measures must be taken to prevent the endemic passage of the disease into the region and prevent the occurrence of other outbreaks. The only effective way to prevent brucellosis in humans to eliminate the animal reservoir [33]. This requires an integrated approach involving both human health services and veterinary services [28]. Some authors emphasize the role of active serological surveillance and slaughter of infected animals to control disease [34]. Others point to the need for livestock vaccination to effectively prevent animal and human brucellosis [36]. The disease is rare in industrialized countries because of the routine screening and vaccination programs for livestock and domestic animals [36]. Our results confirm the need to educate the public about the risks associated with the consumption of raw milk and dairy products as well as the need for educational programs targeting farmers.

Several studies highlight the importance of isolating, identifying, and genotyping the *Brucella* subspecies to assist in identifying the source of infection and to implement control measures [37]. One limitation of our study was the lack of bacteriological evaluation for all patients, given the difficulty accessing a level 3 biosafety laboratory in our region. Molecular evaluations were also not done.

In conclusion, our study showed that the source of this outbreak, which had not been previously recorded in the Douz district, was foodborne. Consumption of raw milk from smuggled sick goats was the main risk factor. The source farm of the outbreak was disinfected, the sick goats were isolated for slaughter, and the other animals were put under surveillance. Moreover, a health awareness and education program for the public and breeders has been launched. In addition to serological surveillance the slaughter of infected animals, vaccinations, control of animal movements across borders, and control of goat milk sales must be strengthened in order to prevent possible spread of the disease in the southwestern region of Tunisia.

## Figures and Tables

**Figure f3-epih-44-e2022048:**
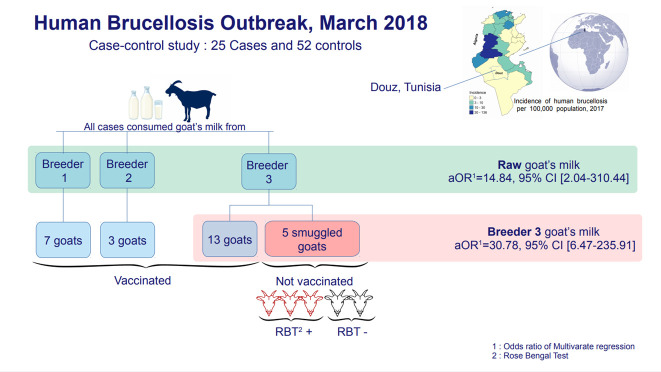


**Table 1.** Age and sex distribution of human brucellosis cases and controls, Douz, Tunisia, March 2018

Variables	Cases (n=25)	Controls (n=52)	Total
Sex			
Male	11 (44.0)	15 (28.8)	26 (33.8)
Female	14 (56.0)	37 (7.1)	51 (66.2)
Age (yr)			
0-14	5 (20.0)	6 (11.5)	11 (14.3)
15-44	10 (40.0)	22 (42.3)	32 (41.6)
45-91	10 (40.0)	24 (46.1)	34 (44.2)

Values are presented as number (%).

**Table 2.** Univariate and multivariate analysis of risk factors for human brucellosis, Douz, Tunisia, March 2018

Risk factors	Cases (n=25)	Controls (n=52)	Crude	Adjusted
Consumption of AZ goat milk	23 (92.0)	9 (17.3)	54.94 (10.94, 275.89)	30.78 (6.47, 235.91)
Raw	23 (92.0)	5 (9.6)	108.10 (19.47, 600.07)	NA
Boiled	0 (0.0)	4 (76.9)	NA	NA
Consumption of goat milk in general	24 (96.0)	21 (40.4)	35.43 (4.45, 282.36)	14.84 (2.04, 310.44)
Contact with AZ goats	5 (20.0)	1 (1.9)	12.75 (1.40, 116.04)	1.39 (0.17, 29.85)
Contact with goats in general	11 (44.0)	14 (26.9)	2.13 (0.78, 5.79)	NA
Sex, male	11 (44.0)	15 (28.8)	1.94 (0.19, 1.39)	NA

Values are presented as number (%) or odds ratio (95% confidence interval). AZ, breeder who sold the milk of his goats door-to-door; NA, not applicable.
